# Nitric Oxide Disrupts Zinc Homeostasis in Salmonella enterica Serovar Typhimurium

**DOI:** 10.1128/mBio.01040-18

**Published:** 2018-08-14

**Authors:** Elaine R. Frawley, Joyce E. Karlinsey, Anshika Singhal, Stephen J. Libby, Paschalis-Thomas Doulias, Harry Ischiropoulos, Ferric C. Fang

**Affiliations:** a Department of Laboratory Medicine, University of Washington, Seattle, Washington, USA; b Department of Microbiology, University of Washington, Seattle, Washington, USA; c Department of Pediatrics, The Children’s Hospital of Philadelphia Research Institute and The Perelman School of Medicine, University of Pennsylvania, Philadelphia, Pennsylvania, USA; National Institute of Child Health and Human Development (NICHD)

**Keywords:** *Salmonella*, nitric oxide, pathogenesis, transporters, zinc homeostasis

## Abstract

Nitric oxide (NO·) produced by mammalian cells exerts antimicrobial actions that result primarily from the modification of protein thiols (*S*-nitrosylation) and metal centers. A comprehensive approach was used to identify novel targets of NO· in Salmonella enterica serovar Typhimurium (*S.* Typhimurium). Newly identified targets include zinc metalloproteins required for DNA replication and repair (DnaG, PriA, and TopA), protein synthesis (AlaS and RpmE), and various metabolic activities (ClpX, GloB, MetE, PepA, and QueC). The cytotoxic actions of free zinc are mitigated by the ZntA and ZitB zinc efflux transporters, which are required for *S.* Typhimurium resistance to zinc overload and nitrosative stress *in vitro*. Zinc efflux also ameliorates NO·-dependent zinc mobilization following internalization by activated macrophages and is required for virulence in NO·-producing mice, demonstrating that host-derived NO· causes zinc stress in intracellular bacteria.

## IMPORTANCE

Nitric oxide (NO·) is produced by macrophages in response to inflammatory stimuli and restricts the growth of intracellular bacteria. Mechanisms of NO·-dependent antimicrobial actions are incompletely understood. Here, we show that zinc metalloproteins are important targets of NO· in *Salmonella*, including the DNA replication proteins DnaG and PriA, which were hypothesized to be NO· targets in earlier studies. Like iron, zinc is a cofactor for several essential proteins but is toxic at elevated concentrations. This study demonstrates that NO· mobilizes free zinc in *Salmonella* and that specific efflux transporters ameliorate the cytotoxic effects of free zinc during infection.

## INTRODUCTION

Nitric oxide (NO·) is generated by the inducible nitric oxide synthase (iNOS) of phagocytic cells during infection. Sustained production of NO^.^ restricts the replication of intracellular bacteria, and iNOS deficiency increases host susceptibility to infection (1, 46). NO· and related reactive nitrogen species (RNS) covalently modify thiols, tyrosine residues, metal centers, nucleotides, and lipids to impair bacterial growth and modulate bacterial virulence (2, 3, 7, 48–50). *S*-Nitrosylation is a reversible thiol modification that can disrupt protein function, sometimes by interfering with disulfide bonding or mobilizing metal cofactors. Although some examples of *S*-nitrosylation have been demonstrated, the full extent of bacterial proteins subject to *S*-nitrosylation and the protein targets responsible for the antimicrobial actions of NO· are incompletely understood.

Zinc metalloproteins typically comprise 3 to 5% of the bacterial proteome (4, 51). A previous study has shown that intracellular zinc can be mobilized during treatment of bacterial cells with an NO· donor (5). Zinc is typically coordinated by either histidine or cysteine residues, the latter representing potential targets of *S*-nitrosylation (6, 7). In Salmonella enterica serovar Typhimurium, intracellular zinc is tightly restricted by two zinc-sensing transcriptional regulators and multiple uptake and efflux transport systems. *S.* Typhimurium acquires zinc from the environment via the high-affinity ZnuABC system and the lower-affinity ZupT transporter (8, 52). Expression of *znuABC* is regulated by Zur, a member of the Fur family of bacterial regulators (8, 53–55). Under zinc-replete conditions, Zur binds to zinc in the cytoplasm and represses *znuABC* transcription. Under low-zinc conditions, apo-Zur is incapable of DNA binding, and the repression of *znuABC* transcription is relieved. The regulation of *zupT* expression is uncharacterized and may be constitutive (9). Cells typically maintain intracellular free zinc at picomolar to femtomolar levels (10, 11). As free zinc levels rise, zinc is bound by the transcriptional activator ZntR, which activates transcription of the high-affinity efflux transporter ZntA (12, 56, 57). Additional efflux transporters, called ZntB, ZitB, and YiiP, have been described in Escherichia coli and *S.* Typhimurium (13, 21, 22, 58). Recent work has suggested that ZitB acts in conjunction with ZntA to protect *S.* Typhimurium from zinc overload, but the contribution of these transport systems in maintaining zinc homeostasis during infection *in vivo* and the mechanisms by which their expression is regulated have yet to be fully elucidated (14).

The ZnuABC and ZupT zinc uptake systems are required for full virulence of *S.* Typhimurium (15, 59–61). However, zinc efflux has only recently been implicated in *Salmonella* virulence (14). We hypothesized that zinc mobilized from metalloproteins by phagocyte-derived NO· might be exported by zinc efflux systems to mitigate the consequences of zinc stress during infection. Here, we show that *Salmonella* zinc metalloproteins are targets of *S*-nitrosylation, and zinc efflux is required for *Salmonella* resistance to nitrosative stress both *in vitro* and in a murine model of infection.

## RESULTS

### The *S*-nitrosoproteome of *S*. Typhimurium includes proteins involved in essential cellular functions.

**FIG 1:**
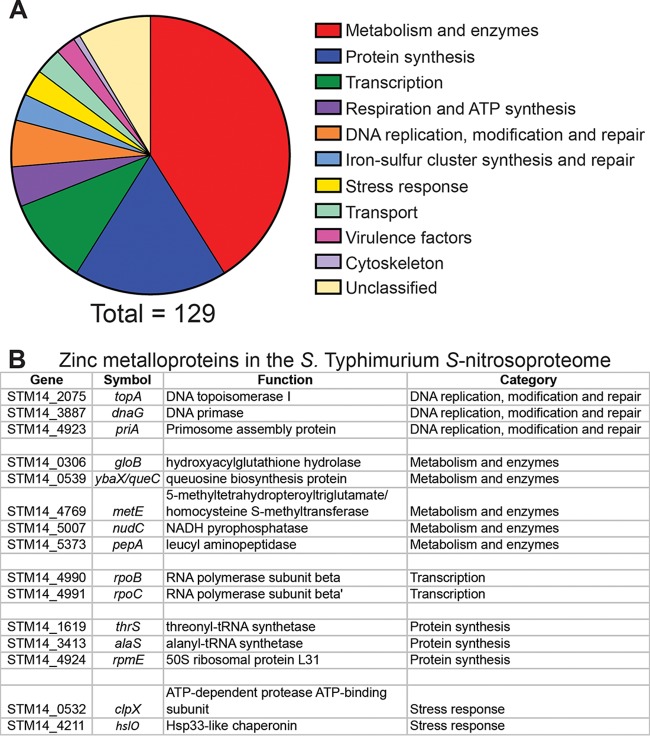
Classification of proteins in the *S.* Typhimurium *S*-nitrosoproteome. (A) Functional classification of proteins with cysteine residues modified by NO· treatment. A total of 141 modified proteins were identified, and classification is shown as a percentage of the total. (B) Of the proteins modified by NO·, 15 (~10%) were found to be zinc metalloproteins. The metalloproteins are sorted by functional category and listed in numerical order by gene identifier in *S.* Typhimurium strain 14028s.

To identify *S.* Typhimurium proteins susceptible to *S*-nitrosylation, cell lysates were treated with the NO· donor diethylamine (DEA) NONOate (DEANO; half-life, 2 min), which generates a rapid bolus of NO·, for 10 min. *S*-nitrosylated proteins were selectively enriched by binding to a phenyl mercury column. Following on-column digestion with trypsin, 144 peptides from 129 proteins were identified by mass spectrometry (MS). The full list of *S*-nitrosylated peptides can be found in Table S1 in the supplemental material. The identified proteins are representative of a wide variety of functional categories, including transcription, respiration, iron-sulfur cluster metabolism, stress responses, and DNA replication or repair, with the majority consisting of enzymes or proteins involved in protein synthesis (Fig. 1A). Thirty-three of the targets are categorized as essential proteins in either *S*. Typhimurium or E. coli (16, 62, 63). Previously identified targets of *S*-nitrosylation, such as OxyR, LpdA, and FabB, were identified, along with many novel targets (17, 64, 65). To verify one of these novel targets, the enzymatic activity of GlyA (serine hydroxymethyltransferase [SHMT]) was assayed in cell lysates treated with either DEANO or DEA as a control. *In vitro* GlyA activity was monitored by measuring optical density at 420 nm (OD_420_) to determine the concentration of formaldehyde consumed. Lysates treated with DEANO displayed a greater than 50% reduction in GlyA activity compared to DEA-treated lysates, confirming that GlyA is a novel target of nitric oxide inhibition (Fig. S1).

10.1128/mBio.01040-18.1 FIG S1  GlyA enzymatic activity is inhibited by NO·. *S.* Typhimurium expressing GlyA from a plasmid was treated with 10 mM diethylamine (DEA) as a mock treatment or 10 mM diethylamine NONOate (DEANO). Cell lysates were assayed for serine hydroxymethyltransferase activity by monitoring the glycine-dependent reduction of formaldehyde using a spectrophotometer at 420 nm. The specific activities of the DEANO-treated lysates were normalized to the activities of the corresponding mock DEA-treated lysate. Data are presented as percent specific activity compared to mock treatment. Error bars represent standard deviation from the mean for three biological replicates. Each biological replicate was analyzed in triplicate. The asterisk indicates statistical significance of *P* = 0.01 by paired two-tailed *t* test. Download FIG S1, TIF file, 0.2 MB. Copyright © 2018 Frawley et al. 2018 Frawley et al. This content is distributed under the terms of the Creative Commons Attribution 4.0 International license.

10.1128/mBio.01040-18.8 TABLE S1  Peptides identified in the analysis of the *S*-nitrosoproteome of *S.* Typhimurium. Download TABLE S1, XLS file, 0.1 MB. Copyright © 2018 Frawley et al. 2018 Frawley et al. This content is distributed under the terms of the Creative Commons Attribution 4.0 International license.

### NO· targets zinc metalloproteins and disrupts zinc homeostasis.

**FIG 2:**
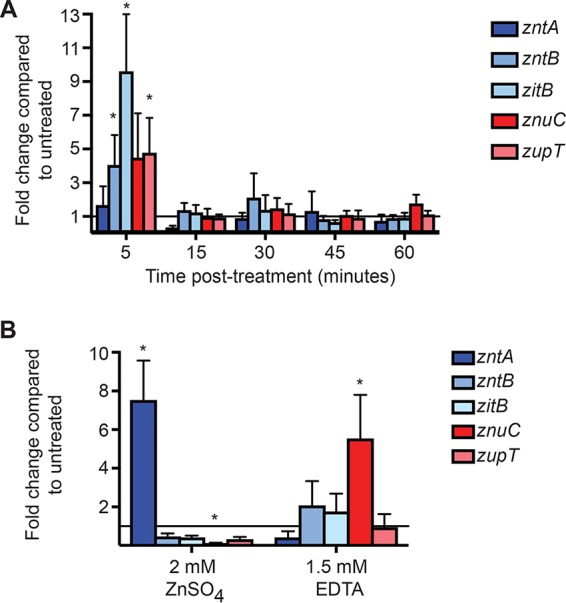
Expression of zinc transport systems in *S.* Typhimurium. qPCR data are presented as a positive fold change of treated compared to untreated cells with zinc efflux systems shown in blue and zinc acquisition systems shown in red. The solid line indicates a fold change of 1 to delineate between upregulation (>1) and downregulation (<1). (A) At 5 min after treatment with the NO· donor diethylamine NONOate (DEANO), expression of all zinc transport systems except *zntA* (dark blue bar) was modestly upregulated. The expression changes for *zntB*, *zitB*, and *zupT* achieved statistical significance with *P* values of 0.05, 0.004, and 0.03, respectively. At later time points, there was no significant difference in zinc transporter expression between treated and untreated cells. (B) In the presence of excess zinc (2 mM ZnSO_4_), expression of the high-affinity zinc efflux system *zntA* (dark blue bar) was significantly upregulated (*P* = 0.04), while expression of the high-affinity acquisition system *znuABC* (represented by *znuC*, red bar) was downregulated (*P* = 0.005). When cultures were treated with 1.5 mM EDTA to chelate zinc in the medium, expression of the *znuABC* acquisition system increased (*P* < 0.001). *, statistical significance was determined by one-sample *t* test compared to theoretical means of 2 for upregulated genes and 0.5 for downregulated genes. Data are the means from 3 (ZnSO_4_) and 8 (EDTA) replicates. Error bars represent standard deviations.

A significant number of zinc metalloproteins were shown to be *S*-nitrosylated by NO· (Fig. 1B). These include DnaG and PriA, which were previously suggested to be targets of NO· inhibition but not experimentally verified (5). As the modification of zinc-coordinating cysteine ligands would be anticipated to disrupt metal binding and release free zinc, we examined the effects of NO· on the expression of zinc transport systems (Fig. 2A). The transcriptional regulators ZntR and Zur sense and respond to altered levels of intracellular free zinc to regulate expression of *zntA* and *znuABC*, respectively (11, 18, 55, 66).

Expression of genes encoding three zinc efflux systems, *zntA*, *zntB*, and *zitB* (Fig. 2A, blue), and two zinc acquisition systems, *znuABC* and *zupT* (Fig. 2A, red), was monitored by quantitative PCR (qPCR) following treatment with 2 mM DEANO for 1 h. At 5 min posttreatment, transcript levels of both zinc uptake and export genes, with the exception of *zntA*, were elevated (Fig. 2A). By 15 min posttreatment, expression returned to a level that was not significantly different from untreated cells and subsequently remained constant for 60 min. Given the increased expression of all transport systems except ZntA, and the transient nature of expression in response to NO·, it is possible that qPCR was insufficiently sensitive to detect *zntA* expression. To investigate this possibility, the promoter regions of the *S.* Typhimurium *hmp* and *zntA* genes were fused to green fluorescent protein (GFP), and fluorescence was monitored by flow cytometry. Expression of Hmp (flavohemoglobin), an NO·-detoxifying enzyme regulated by the NO·-sensing transcriptional repressor NsrR, was monitored as a control (19). A significant increase in mean GFP intensity in response to NO· treatment was observed at all time points from both the *hmp* and *zntA* promoters (Fig. S2). Expression of the zinc transport systems in response to zinc supplementation or the metal chelator EDTA was as expected (Fig. 2B), with *zntA* expression (dark blue bars) increased in the presence of zinc and reduced in the presence of EDTA, whereas *znuC* expression (dark red bars) exhibited the opposite pattern. Expression of *zntB*, *zitB*, or *zupT* did not change significantly under either condition. Although the regulation of *zntB* expression has not been characterized, these results are consistent with expression patterns observed for *zitB* and *zupT* in E. coli (9, 20).

10.1128/mBio.01040-18.2 FIG S2  Mean fluorescence of GFP expressed from the *S.* Typhimurium *hmp* and *zntA* promoters increases in response to NO·. *S.* Typhimurium containing a plasmid expressing GFP from either the *hmp* (A) or *zntA* (B) promoter was treated with 2 mM DEANO and compared to untreated cells by flow cytometry at 5, 15, 30, 45, and 60 min. Mean fluorescence intensity increased in response to NO· for both promoter constructs at all times posttreatment, indicating that GFP expression occurred. Data are the means from three biological replicates, and error bars represent standard deviations. (A) An asterisk indicates statistical significance of *P* = 0.002 (5 min) or *P* < 0.001 (15, 30, 45, and 60 min) by unpaired two-tailed *t* test. (B) An asterisk indicates statistical significance of *P* = 0.04 (5 min), *P* = 0.009 (15 min), and *P* < 0.001 (30, 45, and 60 min) by unpaired two-tailed *t* test. Download FIG S2, TIF file, 0.8 MB. Copyright © 2018 Frawley et al. 2018 Frawley et al. This content is distributed under the terms of the Creative Commons Attribution 4.0 International license.

**FIG 3:**
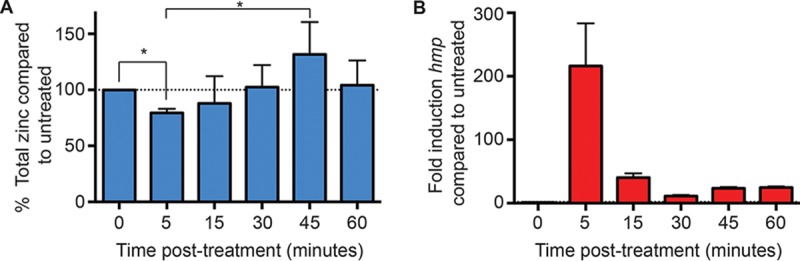
ICP-MS analysis of total cellular zinc content in *S.* Typhimurium following NO· treatment and transcriptional monitoring of NO· sensed by cells. (A) *S.* Typhimurium cells at an OD_600_ of ≈1 were treated with 2 mM diethylamine NONOate (DEANO), and total cellular zinc was measured at various times posttreatment. By 5 min posttreatment, total cellular zinc had fallen significantly compared to untreated cells, suggesting that zinc is effluxed from the cell following NO· treatment. Zinc levels gradually recovered to baseline levels over the course of 60 min. Statistical significance was determined by unpaired two-tailed *t* test; * indicates *P* values of <0.001 and 0.036, respectively. Error bars represent standard deviations. (B) A portion of each culture at each time point was also used to prepare RNA and cDNA for transcriptional analysis. Data are presented as the mean fold change in transcript level compared to untreated cells at each time point. The error bars represent standard deviations. Expression of *hmp* is regulated by the NO·-sensing NsrR regulator. The high level of expression at 5 min posttreatment indicates that the highest levels of NO· were present during this time period. At later time points, the level of *hmp* transcript declined significantly, indicating that the amount of NO· declined by 15 min posttreatment and remained low thereafter.

After observing the simultaneous expression of zinc uptake and efflux systems in response to NO· treatment, the effect of NO· on total cellular zinc levels was determined by inductively coupled plasma mass spectrometry (ICP-MS). Within 5 min after treatment with the NO· donor DEANO, total cellular zinc levels dropped by about 20% compared to untreated cells (Fig. 3A). The level of total cellular zinc subsequently recovered, exceeding initial levels at 45 min posttreatment before returning to pretreatment levels by approximately 60 min. This brief accumulation of excess zinc prior to restoration of homeostasis may be due to the transient expression of zinc uptake systems also observed in response to NO· (Fig. 2A). To correlate these changes in cellular zinc levels with NO· levels, expression of the NO·-induced *S.* Typhimurium *hmp* gene, a reporter for nitrosative stress, was monitored in parallel (Fig. 3B) (19). Maximum *hmp* expression was observed 5 min posttreatment, suggesting that maximal NO· exposure also occurred within 5 min of DEANO treatment. The subsequent reduction in *hmp* expression at later time points indicates that NO· levels rapidly declined below the threshold required for restoration of NsrR-mediated repression. Total zinc levels began to recover at the same time that *hmp* expression declined. Together, these results suggest that NO· rapidly mobilizes zinc from metalloproteins and that zinc homeostasis is preserved by the excretion of this free zinc in response to nitrosative stress. Once NO· has been detoxified, zinc is reacquired from the environment until baseline levels are restored.

### ZntA and ZitB are the primary zinc efflux systems in *S*. Typhimurium.

**FIG 4:**
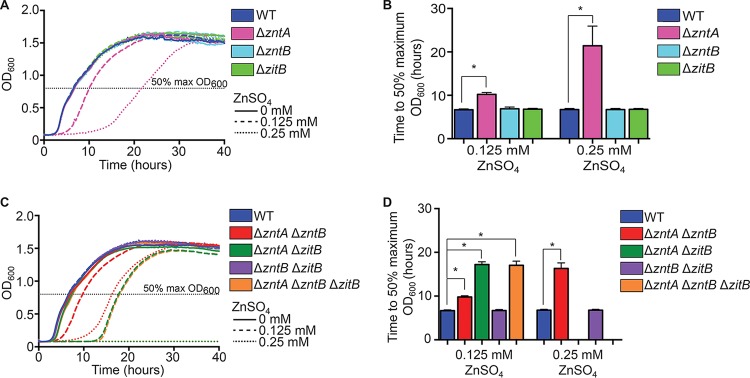
ZntA and ZitB are the primary zinc efflux transporters in *S.* Typhimurium. (A) A Δ*zntA* mutant (pink) was impaired for growth, represented by a delayed exit from lag phase, in both 0.125 mM ZnSO_4_ and 0.25 mM ZnSO_4_, while a Δ*zntB* mutant (aqua) and a Δ*zitB* mutant (light green) exhibited growth comparable to wild type (blue) under these conditions. (B) Significance of the growth defect for the Δ*zntA* mutant in panel A was determined by calculating the mean time required to reach 50% of the maximum final OD_600_ for each strain. (C) A Δ*zntB* Δ*zitB* double mutant (purple) exhibited growth comparable to wild type (blue) when exposed to elevated zinc concentrations. A Δ*zntA* Δ*zntB* mutant (red) behaved similarly to a Δ*zntA* mutant, whereas a Δ*zntA* Δ*zitB* mutant (green) displayed a more severe growth delay at 0.125 mM ZnSO_4_ and was unable to grow at 0.25 mM ZnSO_4_. A Δ*zntA* Δ*zntB* Δ*zitB* triple mutant (orange) exhibited growth characteristics identical to those of a Δ*zntA* Δ*zitB* double mutant. (D) Significance of the growth defects for the mutants in panel C was determined by calculating the mean time required to reach 50% of the maximum final OD_600_ for each strain. Statistical significance of the growth defects was determined by unpaired two-tailed *t* test, and an asterisk indicates *P* values of <0.001 for Δ*zntA*, Δ*zntA* Δ*zntB*, Δ*zntA* Δ*zitB*, and Δ*zntA* Δ*zntB* Δ*zitB* mutants compared to wild type at all concentrations. Error bars represent standard deviations.

*S.* Typhimurium has 3 predicted zinc efflux systems (ZntA, ZntB, and ZitB) as well as a putative fourth system (YiiP), but the relative importance of these systems has not been established. To determine the relative roles played by these systems, mutations in the genes encoding zinc exporters were constructed singly or in combination and assessed for their effects on *S.* Typhimurium tolerance to various zinc concentrations (Fig. 4). Of the single mutants, only the Δ*zntA* mutant (Fig. 4A and B, pink) showed delayed growth in the presence of excess zinc. A Δ*zntA* Δ*zntB* mutant (Fig. 4C and D, red) was no more sensitive than a Δ*zntA* mutant alone. However, a Δ*zntA* Δ*zitB* double mutant was more sensitive to zinc than a Δ*zntA* mutant alone in medium supplemented with 0.125 mM zinc (Fig. 4C and D, green) and was unable to grow at higher concentrations. This indicates that these two efflux systems work cooperatively to protect cells from zinc overload, with ZntA able to compensate for the absence of ZitB but ZitB only partially capable of compensating for the absence of ZntA. A Δ*zntA* Δ*zntB* Δ*zitB* triple mutant (Fig. 4C and D, orange) exhibited susceptibility equivalent to that of a Δ*zntA* Δ*zitB* double mutant. To confirm that the observed mutant phenotypes resulted from the loss of the corresponding zinc efflux transporter, each mutant was complemented by expression in *trans* from its native promoter on a plasmid (Fig. S3). Plasmid-borne ZntA expression was able to fully complement Δ*zntA* or Δ*zntA* Δ*zntB* mutations (Fig. S3A, purple, and B, orange), but *zntB* expression failed to complement a Δ*zntA* Δ*zntB* mutant (Fig. S3B, green). Both the Δ*zntA* Δ*zitB* and Δ*zntA* Δ*zntB* Δ*zitB* mutant strains exhibited the full restoration of zinc tolerance when complemented by expression of either ZntA or ZitB on a plasmid (Fig. S3C, pink and gold, and D, violet and light green). The ability of the ZitB plasmid to fully compensate for the loss of both ZitB and ZntA, whereas chromosomal *zitB* only partially compensates for loss of *zntA*, is likely due to differences in gene dosage.

10.1128/mBio.01040-18.3 FIG S3  Expression of either ZntA or ZitB, but not ZntB, is capable of correcting mutant growth defects in excess zinc. (A and B) The impaired growth of both a Δ*zntA* mutant (pink) and a Δ*zntA* Δ*zntB* mutant (red) can be fully complemented by expression of ZntA from its native promoter on a low-copy-number plasmid (purple [A] or orange [B]), but the growth defect of a Δ*zntA* Δ*zntB* mutant cannot be complemented by expression of ZntB (green [B]). (C and D) Both a Δ*zntA* Δ*zitB* mutant (green [C]) and a Δ*zntA* Δ*zntB* Δ*zitB* mutant (orange [D]) can be fully complemented by heterologous expression of either ZntA (pink [C] and violet [D]) or ZitB (gold [C] and light green [D]). Data are the means from 9 biological replicates from three trials. Statistical significance was determined by unpaired two-tailed *t* test. Download FIG S3, TIF file, 1 MB. Copyright © 2018 Frawley et al. 2018 Frawley et al. This content is distributed under the terms of the Creative Commons Attribution 4.0 International license.

YiiP was initially identified as a putative iron efflux system but later characterized as a transporter of zinc and cadmium (21, 58). A Δ*yiiP* mutant was no more zinc-sensitive than wild-type (WT) *S.* Typhimurium, and Δ*yiiP* Δ*zntA* Δ*zitB* and Δ*yiiP* Δ*zntA* Δ*zntB* Δ*zitB* mutants were no more sensitive than the corresponding double and triple mutant strains, respectively (Fig. S4A and B). These results are consistent with previous observations with Δ*yiiP* mutant derivatives of E. coli and *S.* Typhimurium and suggest that YiiP does not play a role in zinc resistance under these conditions (14, 22).

10.1128/mBio.01040-18.4 FIG S4  YiiP does not contribute to zinc homeostasis in *S.* Typhimurium. (A) A Δ*yiiP* mutant (pink) is no more sensitive to zinc than wild type (wild-type), and both strains grew equally well in the presence or absence of excess zinc. (B) A Δ*zntA* Δ*zitB* Δ*yiiP* strain (purple) is no more sensitive to excess zinc than a Δ*zntA* Δ*zitB* strain (green). A Δ*zntA* Δ*zntB* Δ*zitB* Δ*yiiP* strain (gold) is no more zinc-sensitive than a Δ*zntA* Δ*zntB* Δ*zitB* strain (orange). Together, these results indicate that YiiP does not contribute to zinc homeostasis in *S.* Typhimurium under these experimental conditions, even in the absence of all other known zinc efflux systems. Data are the means from 12 biological replicates from four trials. Statistical significance was determined by unpaired two-tailed *t* test. Download FIG S4, TIF file, 0.7 MB. Copyright © 2018 Frawley et al. 2018 Frawley et al. This content is distributed under the terms of the Creative Commons Attribution 4.0 International license.

### ZntA and ZitB are required for *S*. Typhimurium resistance to nitrosative stress.

**FIG 5:**
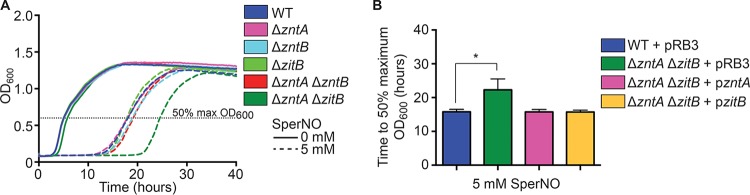
Zinc efflux by ZntA and ZitB is required for *S.* Typhimurium resistance to nitrosative stress *in vitro*. (A) Single zinc efflux mutants (pink, aqua, and light green) and a Δ*zntA* Δ*zntB* double mutant (red) were no more sensitive to NO· generated by the donor spermine NONOate (SperNO) than wild-type cells (blue). However, a Δ*zntA* Δ*zitB* double mutant (green) exhibited significantly delayed growth, indicating enhanced NO· sensitivity (*, *P* < 0.0001). (B) The growth defect of the Δ*zntA* Δ*zitB* mutant was complemented by expression of either ZntA or ZitB from its native promoter in *trans*. Statistical significance was determined by comparing the time required to reach 50% of the maximal OD_600_ (dashed line) by unpaired two-tailed *t* test.

NO· rapidly mobilizes intracellular zinc in *S.* Typhimurium, which is followed by a reduction in total cellular zinc content. We therefore hypothesized that zinc efflux might play an important role in ameliorating the consequences of nitrosative stress. To test this hypothesis, mutant strains lacking zinc efflux transporters singly or in combination were cultured in the presence of the NO· donor spermine NONOate (SperNO; half-life, 39 min), which provides a sustained release of NO· for observation of growth phenotypes. Although none of the single efflux mutants was more sensitive to SperNO than the wild type, a Δ*zntA* Δ*zitB* double mutant exhibited a delayed exit from lag phase, indicative of enhanced sensitivity to NO· (Fig. 5A, dark green dashed line). The reduction in total cellular zinc displayed by wild-type *S.* Typhimurium in response to NO· was not observed in the Δ*zntA* Δ*zitB* double mutant, in which zinc levels remain steady or slightly elevated following NO· treatment (Fig. S5). Together these results indicate that ability to efflux free zinc is an important component of the response to nitrosative stress. The growth defect in the presence of SperNO was complemented by expression of either ZntA or ZitB from its native promoter on a low-copy-number vector (Fig. 5B). The sensitivity of the Δ*zntA* Δ*zitB* double mutant but not a Δ*zntA* single mutant suggests that the amount of zinc mobilized by NO· can be efficiently removed from the cell by ZitB in the absence of ZntA and that the level of zinc stress resulting from 5 mM SperNO was not as great as the zinc stress from the addition of 0.125 mM ZnSO_4_, which impaired the growth of a Δ*zntA* single mutant (Fig. 4A).

10.1128/mBio.01040-18.5 FIG S5  A Δ*zntA* Δ*zitB* mutant does not show a decrease in total cellular zinc following NO· treatment. Δ*zntA* Δ*zitB S.* Typhimurium at an OD_600_ of ≈1 was treated with 2 mM diethlyamine NONOate (DEANO), and total cellular zinc was measured at various times posttreatment by ICP-MS. Total intracellular zinc was not significantly different from baseline (*T*_0_, untreated) at any of the times examined. Download FIG S5, TIF file, 0.8 MB. Copyright © 2018 Frawley et al. 2018 Frawley et al. This content is distributed under the terms of the Creative Commons Attribution 4.0 International license.

### Macrophage-derived NO· induces free zinc accumulation in Δ*zntA* Δ*zitB* mutant *Salmonella*.

A genetically encoded zinc biosensor was used to determine whether macrophage-derived NO· mobilizes zinc in intracellular *Salmonella* and to measure the influence of zinc efflux transporters on zinc accumulation. The ZapCV5 zinc sensor contains the first two zinc fingers of the Zap1 transcription factor from Saccharomyces cerevisiae, coupled to an enhanced cyan fluorescent protein (CFP) fluorescence resonance energy transfer (FRET) donor and a circularly permuted Venus (cp173Venus) FRET acceptor protein (23). Each of the cysteine ligands in the zinc finger region was mutated to histidine in this construct, which only modestly reduces zinc sensitivity and renders the sensor insensitive to *S-*nitrosylation by NO·, which could otherwise confound interpretation of the results. Sensor function was confirmed by adding ZnSO_4_ to the growth medium to drive accumulation of free intracellular zinc and by measuring changes in the FRET ratio. In a Δ*zntA* Δ*zitB* mutant strain, the FRET ratio increased significantly following exposure to increasing ZnSO_4_ concentrations, indicating that absence of these efflux systems results in increased levels of free intracellular zinc, whereas wild-type cells were able to maintain their free zinc pool at a steady level (Fig. S6).

10.1128/mBio.01040-18.6 FIG S6  A fluorescence resonance energy transfer (FRET)-based biosensor for zinc shows accumulation of free intracellular zinc in a Δ*zntA* Δ*zitB* mutant but not in wild-type *S.* Typhimurium. A genetically encoded zinc biosensor, ZapCV5, was expressed constitutively from a plasmid in *S.* Typhimurium. Due to the constant presence of the sensor, a portion of sensor proteins are metallated during normal cell growth. The amount of FRET signal from the sensor was greater in a Δ*zntA* Δ*zitB* mutant than in wild type even when no exogenous ZnSO_4_ (0 mM) was added to the growth medium. At increased concentrations of ZnSO_4_, FRET signal increased in the Δ*zntA* Δ*zitB* mutant but not in the wild-type strain. None of the wild-type FRET ratios were significantly different from one another, whereas the Δ*zntA* Δ*zitB* FRET ratio was significantly greater than the wild-type ratio at each concentration (*P* < 0.001). The FRET ratios for the Δ*zntA* Δ*zitB* mutant at 0.25 mM and 0.5 mM ZnSO_4_ were also significantly elevated compared to the FRET ratio in the absence of exogenous zinc (*P* < 0.001). Statistical significance was analyzed by one-way analysis of variance. Download FIG S6, TIF file, 0.9 MB. Copyright © 2018 Frawley et al. 2018 Frawley et al. This content is distributed under the terms of the Creative Commons Attribution 4.0 International license.

**FIG 6:**
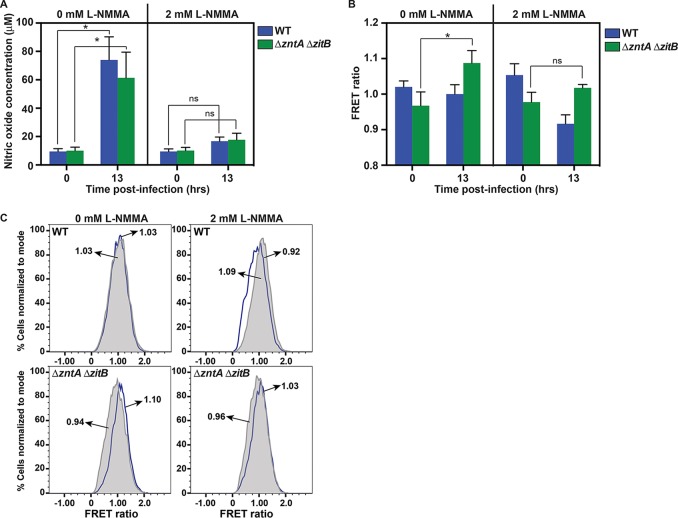
Free intracellular zinc levels increase in a Δ*zntA* Δ*zitB S.* Typhimurium mutant during macrophage infection in response to NO· production. (A) Changes in NO· production are shown at the time of infection (0 h) and 13 h postinfection in the presence or absence of the NOS inhibitor l-NMMA. IFN-γ-primed murine macrophages infected with either wild-type (blue) or Δ*zntA* Δ*zitB* (green) *S.* Typhimurium produced significant levels of NO· after 13 h in the absence of the NOS inhibitor l-NMMA (*P* < 0.001) but not in the presence of 2 mM l-NMMA (ns). (B) Changes in FRET ratio are shown immediately following infection (0 h) and 13 h postinfection. The FRET ratio of the ZapCV5 biosensor increased in Δ*zntA* Δ*zitB* mutants (green) isolated from murine macrophages after 13 h (*P* < 0.001), but not in wild-type *S.* Typhimurium, indicating that intracellular free zinc levels rise in the efflux-deficient mutant during infection. The increase in FRET was not observed when macrophages were treated with 2 mM l-NMMA to inhibit NO· production (ns). (C) Flow cytometry histograms from one representative experiment. The mean value of each histogram (*T*_0_, gray shape, and *T*_13_, blue line) is indicated. Data in panels A and B are presented as the means with error bars representing standard deviations. Statistical significance (*) was determined by one-way analysis of variance.

The zinc biosensor was next used to examine whether NO· production by infected murine macrophages mobilizes free zinc in *S.* Typhimurium. Production of NO· by iNOS peaks hours after infection (46), and a significant increase in nitric oxide was detected after 13 h (Fig. 6A, 0 mM *N*^G^-monomethyl-l-arginine [l-NMMA]). NO· production was inhibited by the addition of 2 mM *N*^G^-monomethyl-l-arginine monoacetate to the cell culture medium (Fig. 6A, 2 mM l-NMMA). In NO·-producing macrophages, an increase in the FRET ratio was observed in the Δ*zntA* Δ*zitB* strain 13 h postinfection but not in wild-type bacteria, in which all zinc efflux systems were functional (Fig. 6B, 0 mM l-NMMA, and C). This FRET ratio increase was abrogated by treatment with the NOS inhibitor l-NMMA. Together, these observations indicate that free intracellular zinc is mobilized from zinc metalloproteins in *S.* Typhimurium by macrophage-derived NO· and is subsequently exported from the cell by ZntA and ZitB. In the absence of ZntA and ZitB, mobilized free zinc remains available to bind to the sensor, leading to an increase in FRET. When NO· production is inhibited by l-NMMA, intracellular free zinc is reduced in both wild-type and mutant cells.

### Zinc efflux is required for *Salmonella* virulence in NO·-producing mice.

**FIG 7:**
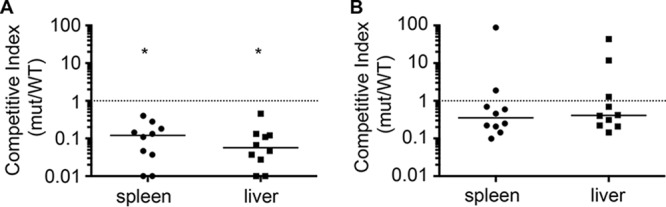
Virulence of Δ*zntA* Δ*zitB* mutant *S.* Typhimurium is attenuated in NO·-producing mice. Solid lines represent the median competitive index (CI) for each organ. The dotted line represents the expected CI if neither strain has a competitive advantage. (A) In wild-type C3H/HeOuJ mice, a Δ*zntA* Δ*zitB* mutant has a significant competitive disadvantage compared to wild type (*P* = 0.002 by Wilcoxon signed-rank test to a hypothetical median of 1 for both spleen and liver). (B) In C3H/HeOuJ mice that cannot produce NO· due to treatment with 500 µg ml^−1^ l-*N*_6_-(1-iminoethyl)lysine dihydrochloride (l-NIL), the mutant no longer has a statistically significant disadvantage compared to wild type, and the CIs are significantly different (*, *P* = 0.007 for spleen and *P* < 0.001 for liver by Mann-Whitney test) from the CI in untreated mice. A total of 10 animals were tested for each condition. Data points at a CI of 0.01 were at the limit of detection for the assay.

As mutant *Salmonella* strains deficient in zinc efflux are more sensitive to nitrosative stress *in vitro* and accumulate intracellular free zinc following internalization by NO·-producing macrophages, we investigated whether zinc efflux is required for *S.* Typhimurium virulence *in vivo* in a murine infection model. Wild-type and Δ*zntA* Δ*zitB S.* Typhimurium strains in a 1:1 ratio were used to infect NO·-producing C3H/HeOuJ mice by intraperitoneal (i.p.) inoculation. Five days postinfection, the output ratios and competitive indexes (CI) were determined for bacteria recovered from the liver and spleen. A Δ*zntA* Δ*zitB* mutant was significantly outcompeted by isogenic wild-type *S.* Typhimurium in both the liver and the spleen (Fig. 7A), indicative of reduced virulence. To determine whether this defect was attributable to the mobilization of zinc by NO·, the infection was repeated in mice treated with the iNOS inhibitor l-*N*_6_-(1-iminoethyl)lysine dihydrochloride (l-NIL) (24). A Δ*zntA* Δ*zitB* mutant no longer exhibited a competitive disadvantage compared to wild-type *S.* Typhimurium in mice treated with l-NIL, indicating that zinc efflux is required for *Salmonella* virulence only in mice capable of NO· production (Fig. 7B).

## DISCUSSION

Nitric oxide (NO·) is an important mediator of the mammalian innate immune response to infection. Modification of protein thiols and metal centers by NO· produces pleiotropic effects on microbial physiology and confers NO· with broad-spectrum antimicrobial activity. Although some direct targets of NO· have been previously identified, many molecular targets of NO· are unknown. Our characterization of the *Salmonella* Typhimurium *S*-nitrosoproteome (see Table S1 in the supplemental material) confirmed several previously identified targets of NO· and identified many novel targets, including proteins with essential functions. Cysteines are known to play structural, metal-coordinating, catalytic, and regulatory roles within proteins; therefore, it is not surprising that a wide variety of targets from different functional categories were identified (25). *S-*Nitrosylation targets surface-exposed cysteines, often flanked by charged residues (26). Although some integral membrane proteins were detected, this category of proteins, along with secreted proteins and those present at low abundance under experimental conditions, is likely to be underrepresented or absent from the data set. Nevertheless, the *S*-nitrosoproteome of *S.* Typhimurium identified in this study provides a foundation for future studies and an opportunity to directly validate new targets of NO·-related antimicrobial activity.

The NO· targets identified in this study include a number of zinc metalloproteins (Fig. 1B), two of which were previously implicated in the inhibition of DNA replication by NO· (5). Many zinc metalloproteins bind metals via cysteine residues, which are no longer able to retain zinc following *S*-nitrosylation. The loss of a zinc cofactor would directly impair protein function, and the release of free zinc into the cytoplasm might also exert broad toxic effects on the cell if the zinc is not promptly removed or bound. Although the precise mechanistic basis of zinc toxicity is not known, it is thought to result, at least in part, from mismetallation of metalloproteins that ordinarily bind other divalent metals (27). Zinc is capable of forming more stable complexes with proteins than metals further down the Irving-Williams series, such as iron and manganese, and might thereby disrupt the function of proteins requiring these cofactors (28). Zinc has also been shown to target and destroy exposed 4Fe-4S clusters of dehydratase enzymes (29).

**FIG 8:**
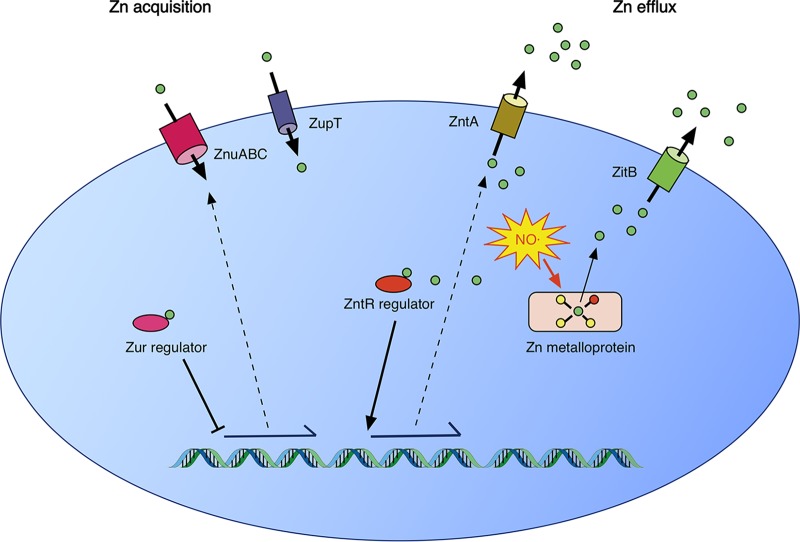
A model of zinc homeostasis in *Salmonella* Typhimurium. Under conditions of zinc deficiency, zinc is not available to bind to the Zur repressor, leading to expression of the ZnuABC zinc acquisition system. ZupT, whose regulation is uncharacterized, has also been shown to contribute to zinc acquisition. When zinc is abundant, Zur bound to zinc represses ZnuABC expression. In addition, free cytoplasmic zinc binds to the transcriptional activator ZntR to induce expression of the ZntA zinc efflux system. Zinc efflux in *S.* Typhimurium is also mediated by ZitB. Under conditions of nitrosative stress, *S*-nitrosylation of cysteine ligands in zinc metalloproteins leads to mobilization of free intracellular zinc. The zinc efflux activities of ZntA and ZitB are required for the resistance of *S.* Typhimurium to nitrosative stress.

*S*-Nitrosylation of zinc metalloproteins was predicted to lead to release of free intracellular zinc, expression of the *zntA* zinc efflux system regulated by ZntR, and repression of the *znuABC* zinc acquisition system regulated by Zur (Fig. 8). However, following NO· treatment, a transient increase in expression of all zinc transporters was observed (Fig. 2A and S2). It is possible that zinc-binding residues within Zur were directly modified by NO·, altering its responsiveness, but these modifications may not have been detected in the *S-*nitrosoproteome assay due to the limited sensitivity of the assay for low-abundance proteins. It is presently uncertain whether NO· leads to dysregulation of transporter expression by direct or indirect actions. Nevertheless, despite the initial dysregulation of transporter expression, total cellular zinc levels were observed to fall immediately following NO· treatment before recovering to baseline levels as NO· levels decreased (Fig. 3).

Of the four putative zinc efflux systems in *S.* Typhimurium, this study demonstrates that ZntA and ZitB are required for resistance to zinc stress, corroborating other recent observations (14). The inducible high-affinity transporter ZntA appears to be the most important of these zinc efflux systems, as an *S.* Typhimurium Δ*zntA* mutant exhibits a growth defect at zinc concentrations that are not inhibitory for a Δ*zitB* mutant strain (Fig. 4). ZntA and ZitB are also required to efflux zinc mobilized by NO· and appear to play complementary roles, as either transporter alone is able to prevent NO· hypersusceptibility (Fig. 5). The mobilization of zinc by NO· and its subsequent efflux by ZntA and ZitB as observed *in vitro* also occur in bacteria following internalization by NO·-producing macrophages (Fig. 6) and during infection of NO·-producing mice, in which a Δ*zntA* Δ*zitB* mutant exhibits a competitive disadvantage in comparison to wild-type *Salmonella* (Fig. 7).

It has been suggested that macrophages may employ zinc as an antimicrobial mediator, which is opposed by the *Salmonella* pathogenicity island 1 (SPI-1) type III secretion system (30, 67). Our observations do not directly address this possibility, but it is nevertheless evident that zinc efflux is required for *Salmonella* virulence and may confer resistance to exogenous zinc as well as to NO·.

Our observations provide new insights into bacterial zinc homeostasis within the host environment. Although most previous studies have focused on the need for bacteria to acquire zinc within the host by competing with the activity of eukaryotic zinc transporters and the chelator calprotectin (31, 59, 68, 69), we have demonstrated that zinc sequestration and removal are also required for pathogenic bacteria to achieve a balance between acquiring zinc for essential cellular functions while minimizing the toxicity of free intracellular zinc mobilized by the actions of NO· on metalloproteins.

## MATERIALS AND METHODS

### Growth conditions.

Salmonella enterica serovar Typhimurium strain ATCC 14028s was used as the wild-type strain for all experiments. *S.* Typhimurium was grown aerobically in Luria-Bertani (LB; Difco) medium at 37°C with shaking at 250 rpm. Antibiotics were used at the following concentrations: 100 µg ml^−1^ ampicillin (Amp), 50 µg ml^−1^ kanamycin (Kan), 20 µg ml^−1^ chloramphenicol (Cm), and 20 µg ml^−1^ tetracycline (Tet).

### Strain and plasmid construction.

All strains are listed in Table S2 in the supplemental material, and primers are listed in Table S3. The Δ*zntA* and Δ*zitB S.* Typhimurium mutant strains were generated using the method of Datsenko and Wanner with the pKD3 Cm or pKD4 Kan cassettes as the templates, and the Δ*yiiP* mutant was generated using the pKD3 template (32). The Δ*zntB* mutant was generated by lambda-red replacement with TetRA insertion (33). Mutations were transduced into a clean ATCC 14028s background using P22 bacteriophage to generate strains EF487 (Δ*zntA*), EF511 (Δ*zntB*), EF512 (Δ*zitB*), and EF561 (Δ*yiiP*). P22 bacteriophage transduction was also used to generate strains EF527 (Δ*zntA* Δ*zntB*), EF528 (Δ*zntA* Δ*zitB*), EF529 (Δ*zntB* Δ*zitB*), and EF530 (Δ*zntA* Δ*zntB* Δ*zitB*). The antibiotic resistance cassettes of these strains were recombinationally excised using pCP20 to generate strains EF531, EF532, EF533, and EF534 (32). Phenotypes of the excised and unexcised strains were identical. To generate strains EF562 and EF563, the Δ*yiiP* mutation was added by P22 bacteriophage transduction into EF532 and EF534. Strain JK377 was derived from strains TH6727 and BC1459 by P22 bacteriophage transduction into JK237 (34).

10.1128/mBio.01040-18.9 TABLE S2  Strains and plasmids used in this study. Download TABLE S2, DOCX file, 0.1 MB. Copyright © 2018 Frawley et al. 2018 Frawley et al. This content is distributed under the terms of the Creative Commons Attribution 4.0 International license.

10.1128/mBio.01040-18.10 TABLE S3  Sequences of primers used in this study. Download TABLE S3, DOCX file, 0.1 MB. Copyright © 2018 Frawley et al. 2018 Frawley et al. This content is distributed under the terms of the Creative Commons Attribution 4.0 International license.

E. coli strain DH10B was used as the host strain for all cloning, and confirmed plasmids were subsequently electroporated into *S.* Typhimurium. The *glyA* gene was cloned into the NheI and HindIII sites of the pBAD18-Cm multiple cloning site (MCS) to generate plasmid pJK715 (35). All zinc mutant-complementing plasmids were generated using the stable pRB3-273C (Amp^r^) plasmid backbone (36). The complementing gene sequences for *zntA*, *zntB*, and *zitB*, including several hundred upstream bases encompassing the native promoter, were cloned into the BamHI and HindIII sites of the pRB3-273C MCS to generate plasmids pJK719, pJK720, and pJK721. To generate pAS3, the ZapCV5 sequence was subcloned from plasmid pcDNA3.1-zapCV5 and inserted into a modified pBAD vector at the BamHI and EcoRI sites (23). Control plasmids pAS4 and pAS5 were created by amplifying and cloning the CFP sequence and cp173Venus sequence into the same modified pBAD vector (23, 37). Plasmids pAS15, pAS16, and pAS17 were generated by amplifying the ZapCV5, CFP, and cp173Venus sequences from plasmids pAS3, pAS4, and pAS5 and cloning into the XbaI and HindIII sites of pHR103 (38). Plasmids pAS20 and pAS22 containing green fluorescent protein (GFP) fused to the promoter regions of the *S.* Typhimurium *hmp* and *zntA* genes, respectively, were generated from source plasmids pJK682 and pRU001. To generate pJK682, the *gfp* gene, without the C-terminal LVA tag, was amplified from plasmid pJBA111 and inserted into the NheI and AatII sites of pBR322 (39, 70). Plasmid pRU001 was created by digesting pJK682 with AvaI, digesting pFPV-mCherry with HindIII, filling cohesive ends using T4 polynucleotide kinase, and digesting both fragments with BamHI before ligating (40). The promoter regions of *hmp* and *zntA* were amplified by PCR and then digested and inserted into the EcoRI site of pRU001 to generate pAS20 and pAS22. Correct promoter orientation was validated by PCR and confirmed by sequencing.

### *S*-Nitrosoproteome analysis.

*S.* Typhimurium was grown overnight in 5 ml LB and then diluted 1:100 into 500 ml fresh LB medium and grown to an optical density at 600 nm (OD_600_) of ~0.5. The cells were pelleted by centrifugation (5,000 × *g*, 5 min, 4°C), washed once in phosphate-buffered saline (PBS), and then resuspended in 3 ml HDN (250 mM HEPES [pH 7.7], 1 mM diethylenetriamine penta-acetic acid [DTPA], 0.1 mM neocuproine), and 1% Triton X-100. The cells were lysed with a French press at 20,000 lb/in^2^ twice. After the first pressing, 150 µl of 25× protease inhibitor cocktail mix (Roche Complete without EDTA) was added. The mixture was clarified by centrifugation at 10,000 × *g* for 15 min at 4°C. The supernatant was passed over a Bio-Gel P-6DG Econo-Pac column (Bio-Rad) equilibrated in 20 ml HDN and 1% Triton X-100 per the manufacturer’s protocol to remove low-molecular-weight thiols. Protein concentration in the eluate was determined using the Coomassie blue protein assay (Thermo Scientific), and 1-ml aliquots were stored at −80°C.

Lysates were diluted to 0.8 mg ml^−1^ in HDN, 1% Triton X-100. Ten milliliters of diluted lysate (~8 mg of total protein) was transferred to 50-ml dark conical tubes (Litesafe II) and treated with either 150 µM diethylamine NONOate (DEANO) or diethylamine (DEA) and incubated at 37°C for 10 min. Thirty milliliters of cold 100% acetone was added and incubated at −20°C for 20 min. The mixture was centrifuged at 3,500 × *g* for 5 min at 4°C, and the pellet was washed in 25 ml cold 75% acetone and centrifuged again. The pellet was resuspended in 10 ml blocking buffer (HDN, 2.5% SDS) and *N*-ethylmaleimide (Sigma-Aldrich) added to 50 mM and incubated at 50°C for 60 min, with vortexing every 5 min to block nonmodified cysteine residues. The proteins were precipitated with acetone as described above and resuspended in 4 ml loading buffer (250 mM 2-(*N*-morpholino)ethanesulfonic acid [MES], pH 6.0, 1 mM DTPA, 1% SDS). A sample was taken to monitor the level of *S*-nitrosylation using the biotin switch method (41).

The lysates were then passed over 4 ml of activated organic mercury resin and washed, and tryptically digested peptides were eluted and analyzed by mass spectrometry essentially as described previously (26).

Data for the DEANO treatment condition were compared to the DEA-only data. Peptides with equal total ion current (TIC) values in the two data sets (TIC DEA/NO − TIC DEA = 0) were discarded from the final analysis. Peptide annotation was performed by using Sorcerer Sequest to search against a UniProt database for *Salmonella* that included methionine dioxidation (+32 Da), cysteine trioxidation (+48 Da), and cysteine *N*-ethylamide alkylation (+125) as variable modifications. Cellular location was predicted using PSORTb (42). Classification as iron or zinc binding was determined by a search of available literature and crystal structures.

### Assay of SHMT activity.

*S.* Typhimurium strains JK1284 and JK1285 containing pBAD18-Cm or pJK715 were grown overnight in LB-Cm and then subcultured 1:100 into 100 ml fresh LB-Cm with 0.2% arabinose and grown to an OD_600_ of 0.8 to 0.9. Cells were pelleted, washed twice in cold 0.85% NaCl, and then resuspended in two 50-ml aliquots. Each cell suspension was sonicated on ice three times for 15 s each at power level 2 with a Misonix Microson Ultrasonic Cell Disrupter XL. Lysates were centrifuged at 4°C to clear cell debris, and supernatants were pooled. Cleared lysates were purified over a Bio-Gel P-6DG Econo-Pac column (Bio-Rad), using the standard protocol to remove low-molecular-weight thiols, and eluted in 2.7 ml 0.85% saline to yield protein concentrations of approximately 4 mg ml^−1^. Two hundred fifty microliters of purified lysate was treated with a 10 mM concentration of either DEA or DEANO for 10 min at 37°C. Serine hydroxymethyltransferase (SHMT) activity assays were carried out according to previously described protocols (43, 44). Briefly, formaldehyde, ditetrahydrofolate, and Nash B reagent were made fresh each time. A 500× pyridoxal 5′-phosphate solution was made once, and aliquots were stored in the dark. Formaldehyde, ditetrahydrofolate, phosphate buffer (pH 7.5), pyridoxal 5′-phosphate, and lysate were combined with or without glycine, and the volume was brought to 1,200 µl with water. The reaction was allowed to proceed for 8 min at 37°C and then stopped by the addition of 300 µl 15% trichloroacetic acid. Samples were centrifuged to remove precipitated protein, and then 500 µl of the supernatant were incubated with 2 ml Nash B reagent for 45 min at 37°C with agitation. SHMT activity was monitored by reading the OD at 420 nm and comparing values to a standard curve generated with known concentrations of formaldehyde. All data were normalized to protein concentration and compared to control reaction mixtures in which glycine was absent. Data are presented as the mean with error bars representing standard deviations. Statistical significance was determined by paired two-tailed *t* test.

### ICP-MS.

An overnight culture of *S.* Typhimurium strain JK377 lacking flagella (for efficient cell pelleting) was subcultured 1:100 into 100 ml fresh LB and grown to an OD_600_ of 1.0. The culture was then divided into 10 6-ml aliquots in glass tubes, and 5 were treated with 2 mM DEANO. At 0, 5, 15, 30, 45, and 60 min posttreatment, 4.5 ml of treated and untreated cultures was pelleted by centrifugation, washed once with 3 ml ultrapure water, and then resuspended in 500 µl analytic-grade nitric acid and incubated in an 85°C water bath for 30 min. The nitric acid solution was diluted 1:10 into MilliQ purified water before inductively coupled plasma-mass spectrometry (ICP-MS) analysis was performed by the Environmental Health Laboratory and Trace Organics Analysis Center at the University of Washington using an Agilent 7500 CE instrument.

### RNA isolation, cDNA synthesis, and gene expression analysis.

Primers for quantitative PCR (qPCR) analysis were published previously (19). For induction by 2 mM DEANO, 1 ml of cells from the same cultures analyzed by ICP-MS was pelleted and resuspended in Trizol reagent. RNA and cDNA were prepared according to previously described protocols (45). qPCR was performed using SYBR green master mix on a Bio-Rad CFX96 real-time system. The *rpoD* gene was amplified for use as an internal control. Statistical significance was determined by one-sample *t* test comparing fold change (expression treated/expression untreated) to theoretical means of 2 for upregulated genes and 0.5 for downregulated genes.

### Flow cytometry-based detection of GFP expression.

Overnight cultures of JK237, AS212, and AS214 were subcultured 1:100 into 25 ml fresh LB and grown to an OD_600_ of 1.0. Cultures were divided into 3-ml aliquots and either treated with DEANO or left untreated. At 5, 15, 30, 45, and 60 min posttreatment, 0.5 ml of culture was pelleted by centrifugation and fixed in 1 ml 2.5% paraformaldehyde for 30 min at 37°C followed by resuspension in 1 ml phosphate-buffered saline (PBS). Fixed bacterial cells were analyzed using an LSR II flow cytometer (Becton, Dickinson). Emission was collected using a 530/30 filter following excitation at 488 nm. Cells were gated according to forward and side scatter (FSC/SSC), and photomultiplier tube voltages were adjusted using wild-type *S.* Typhimurium strain JK237. A total of 10,000 events were collected, and mean fluorescence intensity of GFP was calculated and plotted using FlowJo v10.3 software (TreeStar, Inc.). Data were collected as three biological replicates analyzed on the same day. Statistical significance was determined by unpaired two-tailed *t* test.

### Zinc sensitivity assays.

*S.* Typhimurium wild-type (WT) (JK237) and mutant strains lacking specific zinc efflux pumps (EF487, EF511, EF512, EF531, EF532, EF533, and EF534) were grown overnight in 5 ml LB and then diluted 1:1,000 into fresh LB with or without 0.125 mM or 0.25 mM ZnSO_4_ to a final volume of 300 µl in microtiter plate wells. Cultures were grown aerobically with shaking at 37°C in a Labsystems Bioscreen C machine (Growth Curves USA). Growth was monitored by measuring OD_600_ every 15 min. Differences between cultures were determined by calculating the time to reach 50% maximum OD_600_, and statistical significance was determined by unpaired two-tailed *t* tests. Complementation experiments were conducted as described above using strains JK895, EF535, EF536, EF539, EF540, EF553, EF543, EF544, EF545, EF548, EF549, and EF550.

### Nitric oxide sensitivity assays.

*S.* Typhimurium wild type (JK237) and isogenic zinc efflux-deficient mutant derivatives (EF487, EF511, EF512, EF527, EF528, EF529, and EF530) were grown overnight in 5 ml LB and then diluted 1:1,000 into fresh LB with or without 5 mM SperNO to a final volume of 300 µl in microtiter plate wells. Cultures were grown aerobically with shaking at 37°C in a Labsystems Bioscreen C machine (Growth Curves USA). Growth was monitored by measuring OD_600_ every 15 min. Differences between cultures were determined by calculating the time to reach 50% maximum OD_600_, and statistical significance was determined by unpaired two-tailed *t* tests. Complementation experiments were performed as described above using strains JK895, EF543, EF544, and EF545.

### Macrophage infections.

The murine macrophage-like cell line RAW 264.7 (TIB-71; ATCC) was maintained in Dulbecco’s modified Eagle medium, 4.5 g liter^−1^ d-glucose, 4 mM l-glutamine, 110 mg liter^−1^ sodium pyruvate (DMEM) (Gibco) supplemented with 10% heat-inactivated fetal bovine serum (Fisher Scientific), penicillin, and streptomycin. One day prior to infection, cells were seeded in 24-well plates (5 × 10^5^ cells per well) with DMEM (minus phenol red) supplemented with 200 U ml^−1^ gamma interferon (IFN-γ) (Millipore) and 100 ng ml^−1^ *S.* Typhimurium lipopolysaccharide (Sigma). Nitric oxide production by iNOS was inhibited by addition of 2 mM *N*^G^-monomethyl-l-arginine monoacetate (l-NMMA; AG Scientific). Overnight AS168 and AS172 cultures were harvested, and 0.1 ml of cells was opsonized in mouse serum. Macrophages were infected with opsonized bacteria at a multiplicity of infection (MOI) of 10:1. The plate was spun at 1,000 rpm for 5 min to synchronize infection followed by 20 min of incubation in a CO_2_ incubator. Wells were washed three times with PBS followed by addition of fresh medium containing IFN-γ, l-NMMA, and 20 µg ml^−1^ gentamicin. Three wells each were immediately processed for *T*_0_ samples by lysing macrophages with 1% Triton X-100. After 13 h, the growth medium was collected for analysis of nitric oxide production by the Griess reaction (46). Wells were washed with PBS, and macrophages were lysed with 1% Triton X-100 to collect surviving bacteria. Bacteria from three wells were pooled and fixed for analysis by flow cytometry.

### Detection of free zinc by FRET.

Bacterial cells were fixed in 1 ml 2.5% paraformaldehyde for 30 min at 37°C followed by resuspension in 1 ml phosphate-buffered saline (PBS). Fixed bacterial cells were analyzed using an LSR II flow cytometer (Becton, Dickinson). CFP and fluorescence resonance energy transfer (FRET) signals were detected after excitation at 405 nm, and emission was collected with a 450/50-nm filter for the CFP channel and a 530/30-nm filter for the FRET channel. The cp173Venus protein was excited at 488 nm, and emission was collected with a 530/30-nm filter. Cells were gated according to forward and side scatter (FSC/SSC), and photomultiplier tube voltages were adjusted using the signal from cells expressing only CFP (AS169) or only cp173Venus (AS170) (see Fig. S7 in the supplemental material). Data were processed with FlowJo v10.3 software (TreeStar, Inc.), and FRET ratios for each event were calculated in the double-positive cell population according to the following equation: FRET ratio = [FRET intensity − *a* × (CFP intensity) − *b* × (cp173Venus intensity)]/CFP intensity, where *a* is mean FRET intensity/CFP intensity in cells expressing CFP only and *b* is mean FRET intensity/cp173Venus intensity in cells expressing cp173Venus only. A total of 10,000 events were collected for each experiment.

10.1128/mBio.01040-18.7 FIG S7  Flow cytometry strategy for calculating FRET ratio. (A) Donor (CFP) bleed-through in the FRET channel was calculated as the mean value of FRET intensity/CFP intensity in CFP-expressing cells. Direct excitation of the acceptor (cp173Venus) was calculated as the mean FRET intensity/cp173Venus intensity in cp173Venus-expressing cells. These values were used to calculate the FRET ratio in cells expressing the full biosensor according to the formula listed in Materials and Methods. The images show histograms of Ratio_CFP and Ratio_cp173Venus along with their gating strategies. Live bacteria were gated from forward versus side scatter plots (FSC versus SSC). Live bacteria were further gated based on CFP and cp173Venus intensities. (B) The histograms and gating strategies of control strains used for infecting RAW 264.7 macrophages (AS169, AS170, JK237, and AS172). The FRET ratio was calculated for each event according to the formula presented in Materials and Methods. Histograms of each strain are shown from the cells present in the double-positive gate (CFP+, cp173Venus+). The double-positive gate was created on a CFP-versus-cp173Venus scatter plot. Download FIG S7, TIF file, 1.5 MB. Copyright © 2018 Frawley et al. 2018 Frawley et al. This content is distributed under the terms of the Creative Commons Attribution 4.0 International license.

To validate the response of the zinc FRET biosensor, strains AS168 and AS172 were grown overnight in LB-Amp and diluted to an OD_600_ of 0.2 in 5 ml fresh medium supplemented with ZnSO_4_. Following 30 min of growth, 1 ml of culture was harvested by centrifugation, and cells were fixed for analysis.

### Competitive infections.

All mouse work was approved by the University of Washington Institutional Animal Care and Use Committee (IACUC) and performed according to protocol 3373-01. Ten-week-old female C3H/HeOuJ mice were obtained from The Jackson Laboratory. Three days prior to infection, one group was switched to drinking water containing 500 µg ml^−1^ l-*N*_6_-(1-iminoethyl)lysine dihydrochloride (l-NIL). Bacteria were grown overnight in 5 ml LB and then diluted in PBS. Wild-type and mutant bacteria (JK237 and EF528) were combined to form a 1:1 ratio of 2 × 10^3^ total CFU ml^−1^, and mice were injected intraperitoneally with 500 µl of cells. Quantitative plating followed by selective patching of 100 colonies onto selective plates was used to determine the input CFU and input ratios. Five days postinfection, the mice were euthanized. Livers and spleens were homogenized in PBS, diluted, and plated for enumeration of CFU on LB agar. Colonies were then patched onto appropriate selective medium to determine the output ratio of wild-type to mutant organisms. The output ratio was compared to the input ratio to determine the competitive index (CI) (47). CI is the ratio of (mutant/wild type)_output_ to (mutant/wild type)_input_. A CI of 1 indicates that wild-type and mutant cells have equal fitness during infection, while a CI of <1 indicates that the mutant has a competitive disadvantage. Statistical analysis was performed using the Wilcoxon rank sum test to determine the significance of each CI in GraphPad Prism (version 6).
