# Supplementary figures and images for: Nitric Oxide Disrupts Zinc Homeostasis in Salmonella enterica Serovar Typhimurium
Source: mBio. 2018 Aug 14;9(4):e01040-18. doi: 10.1128/mBio.01040-18 (PMC6094482; doi:10.1128/mBio.01040-18)

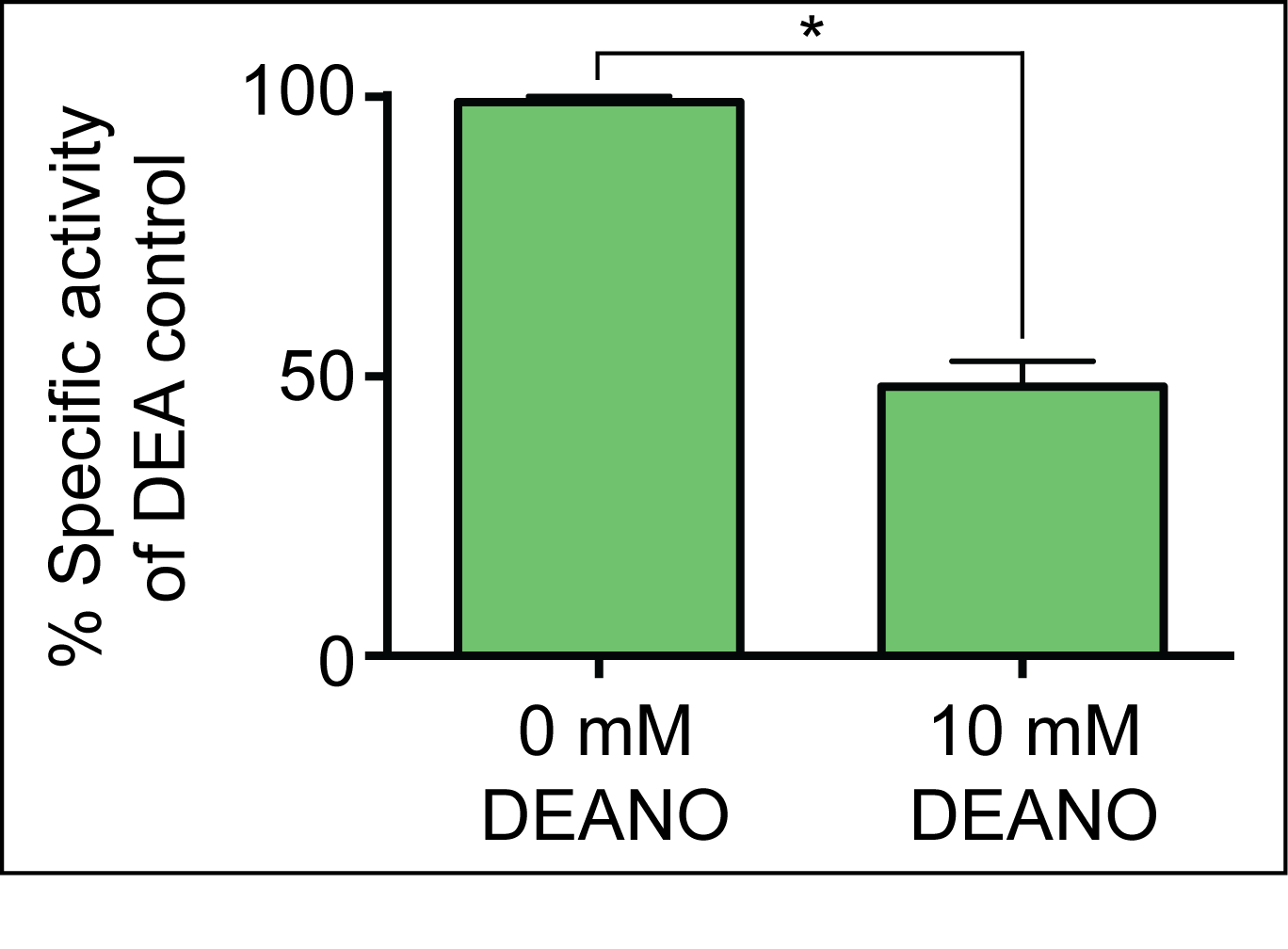

Supplement: FIG S1 [file mbo004184024sf1.tif]

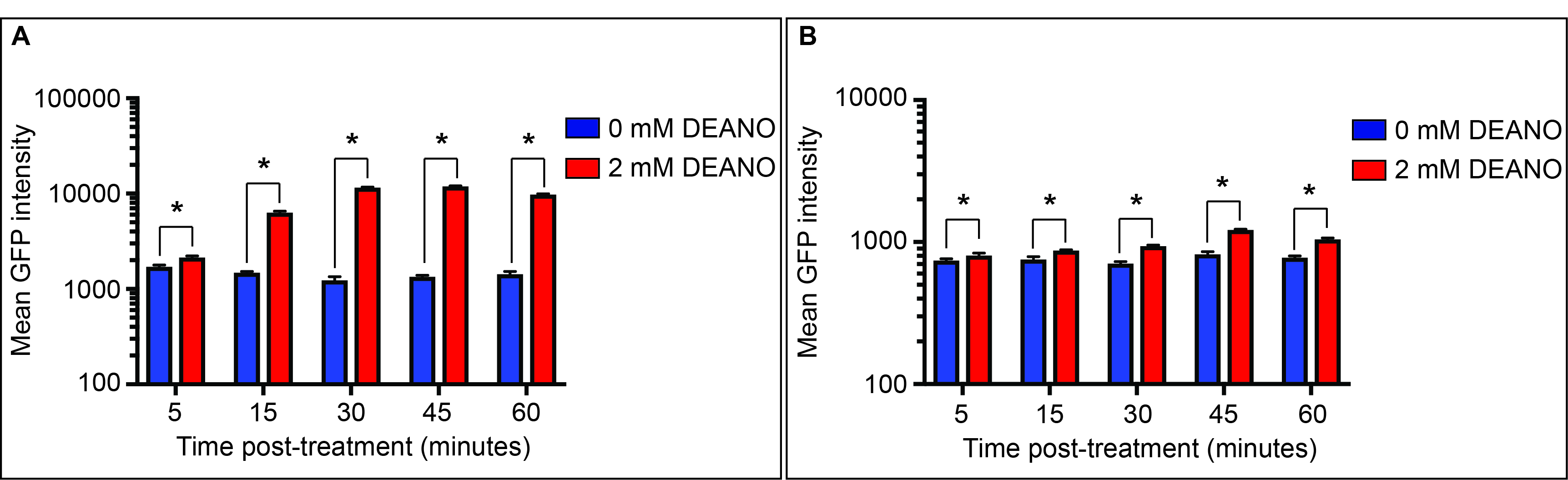

Supplement: FIG S2 [file mbo004184024sf2.tif]

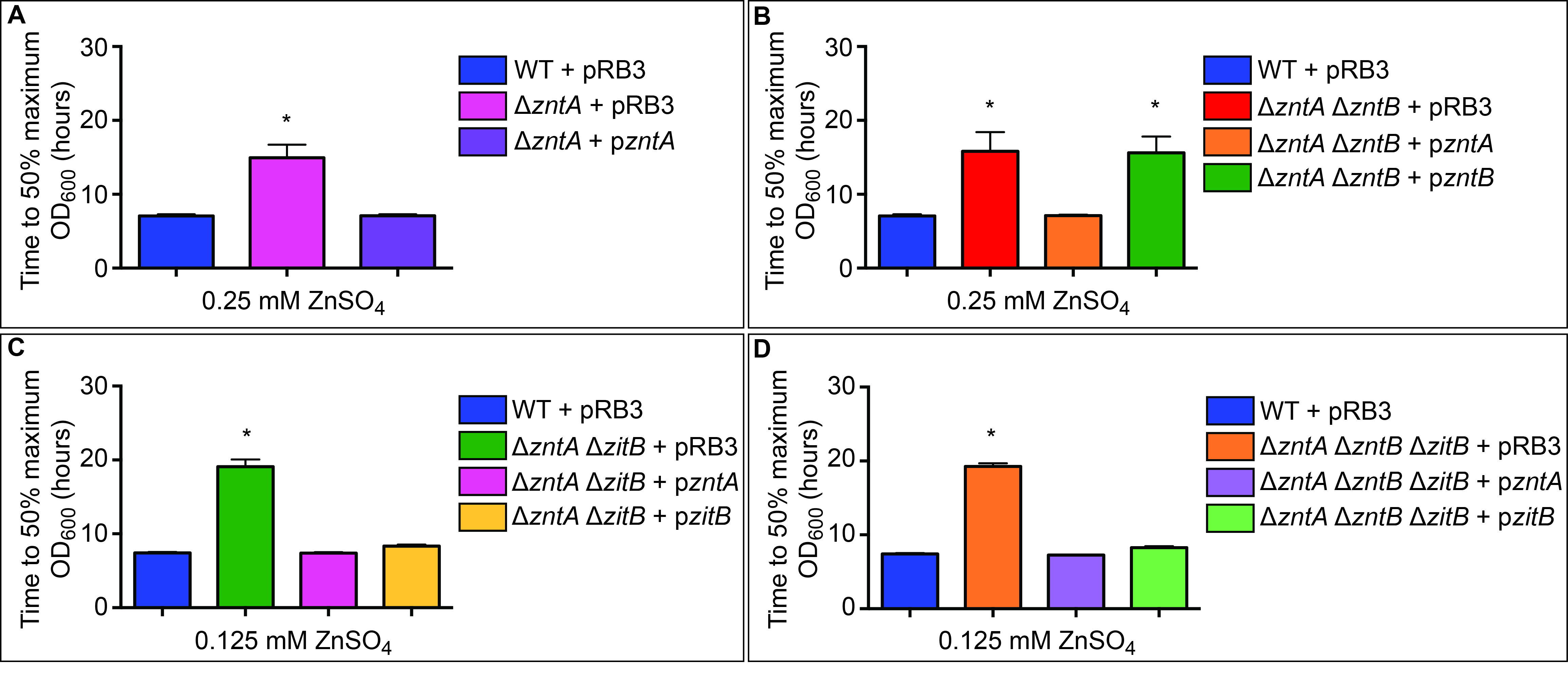

Supplement: FIG S3 [file mbo004184024sf3.tif]

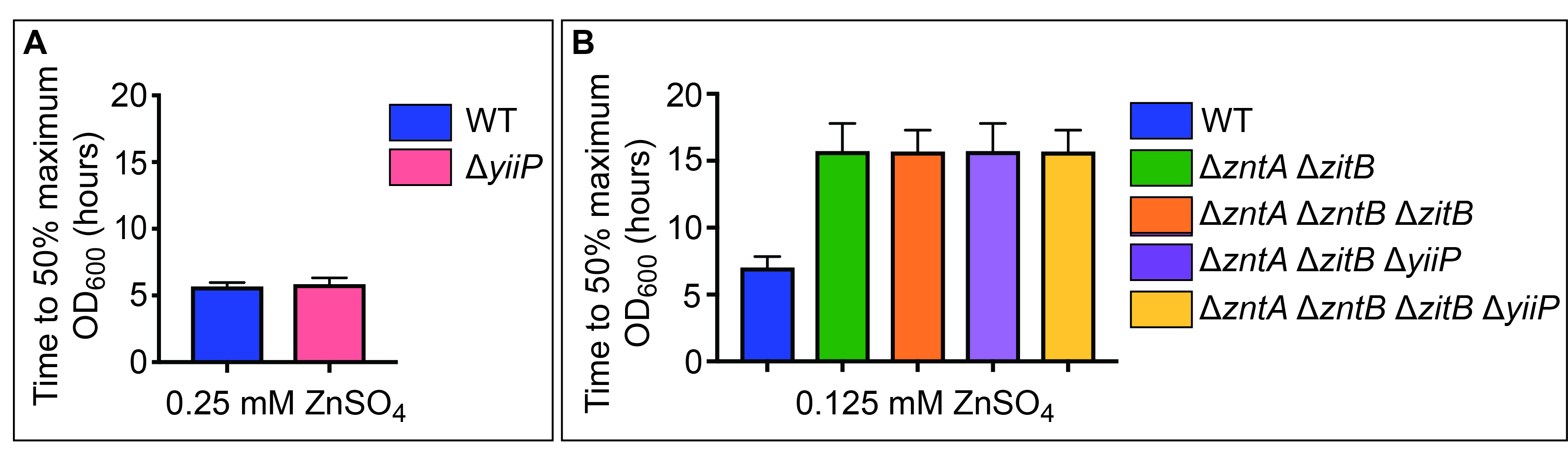

Supplement: FIG S4 [file mbo004184024sf4.tif]

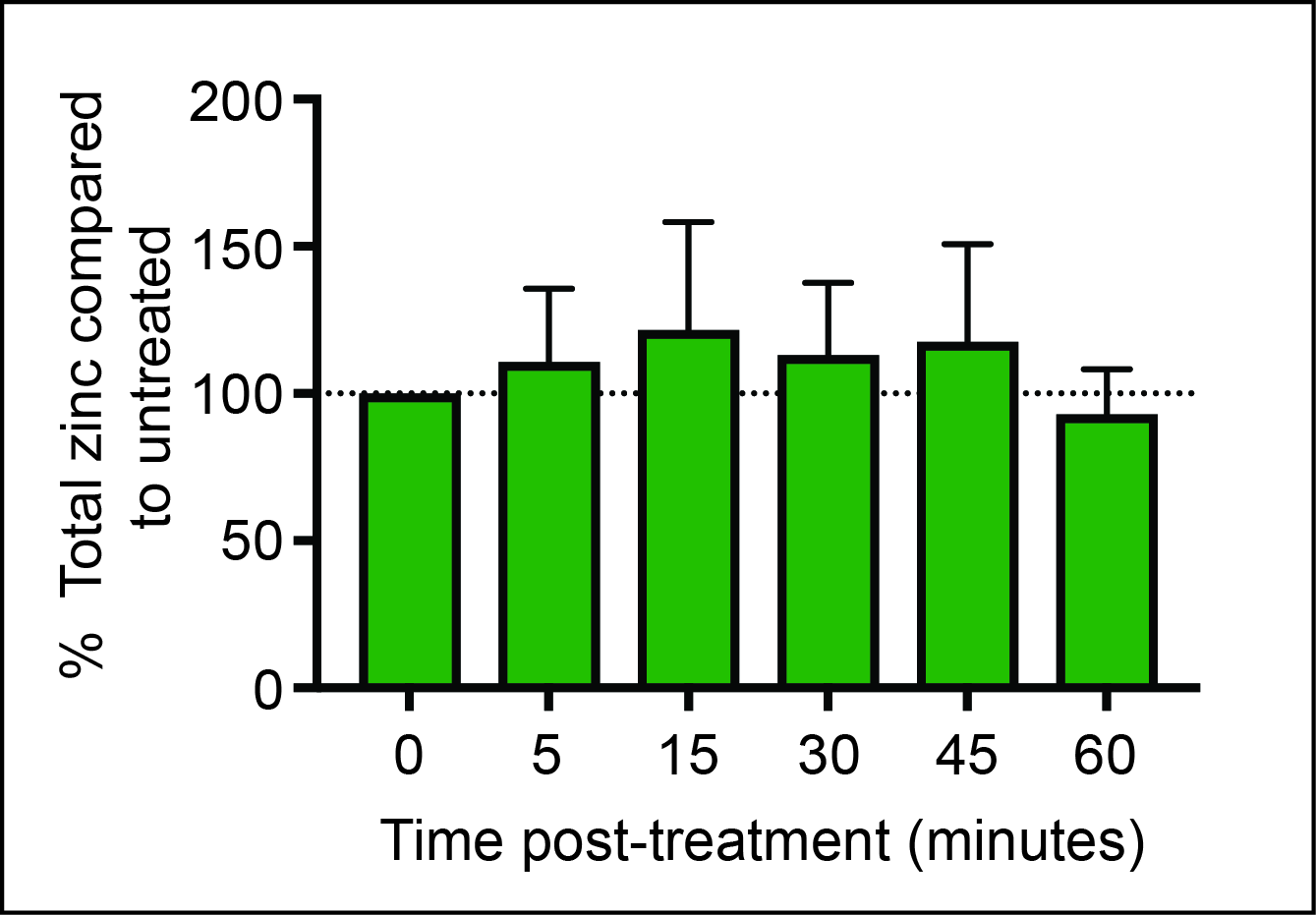

Supplement: FIG S5 [file mbo004184024sf5.tif]

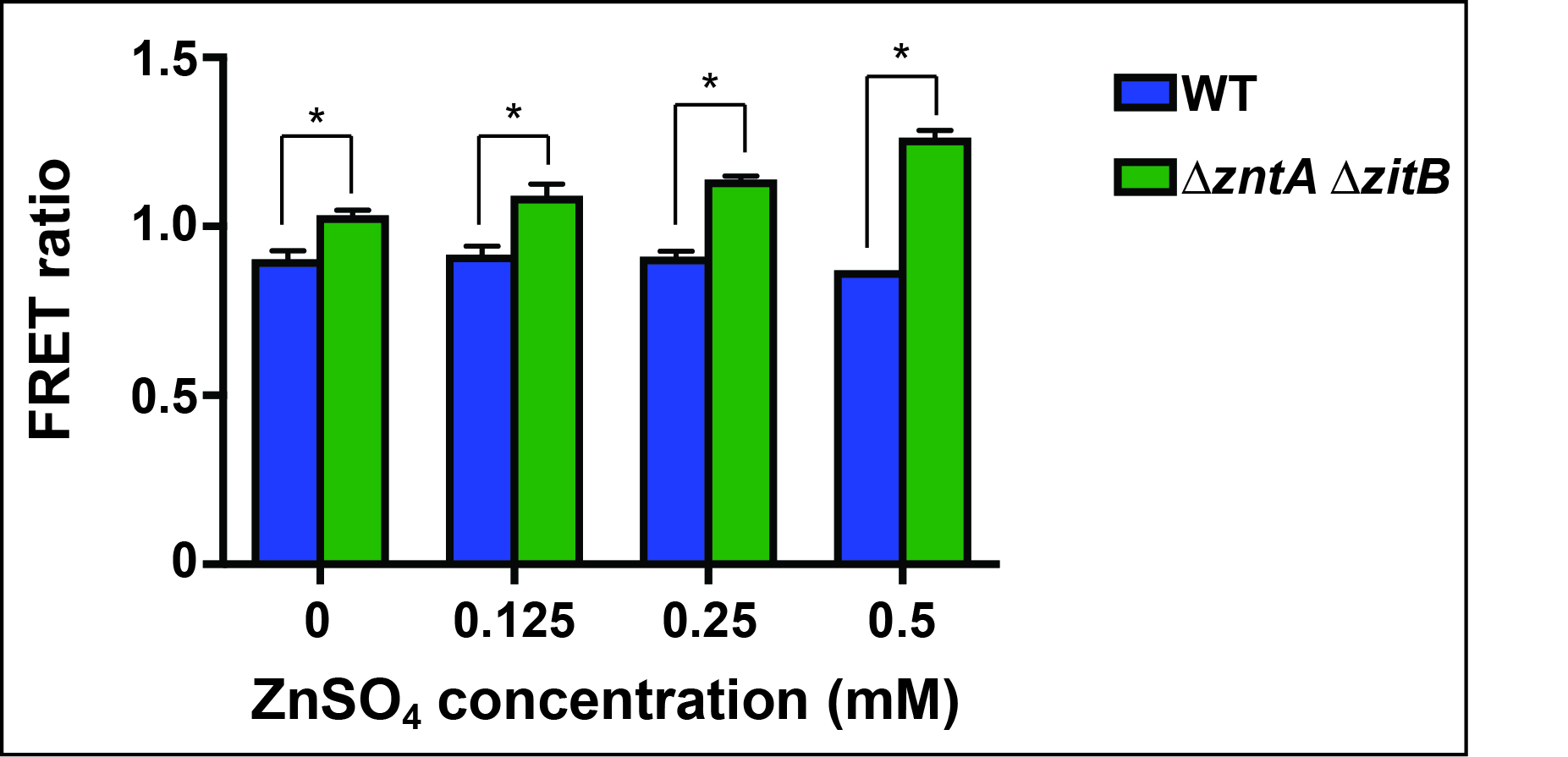

Supplement: FIG S6 [file mbo004184024sf6.tif]

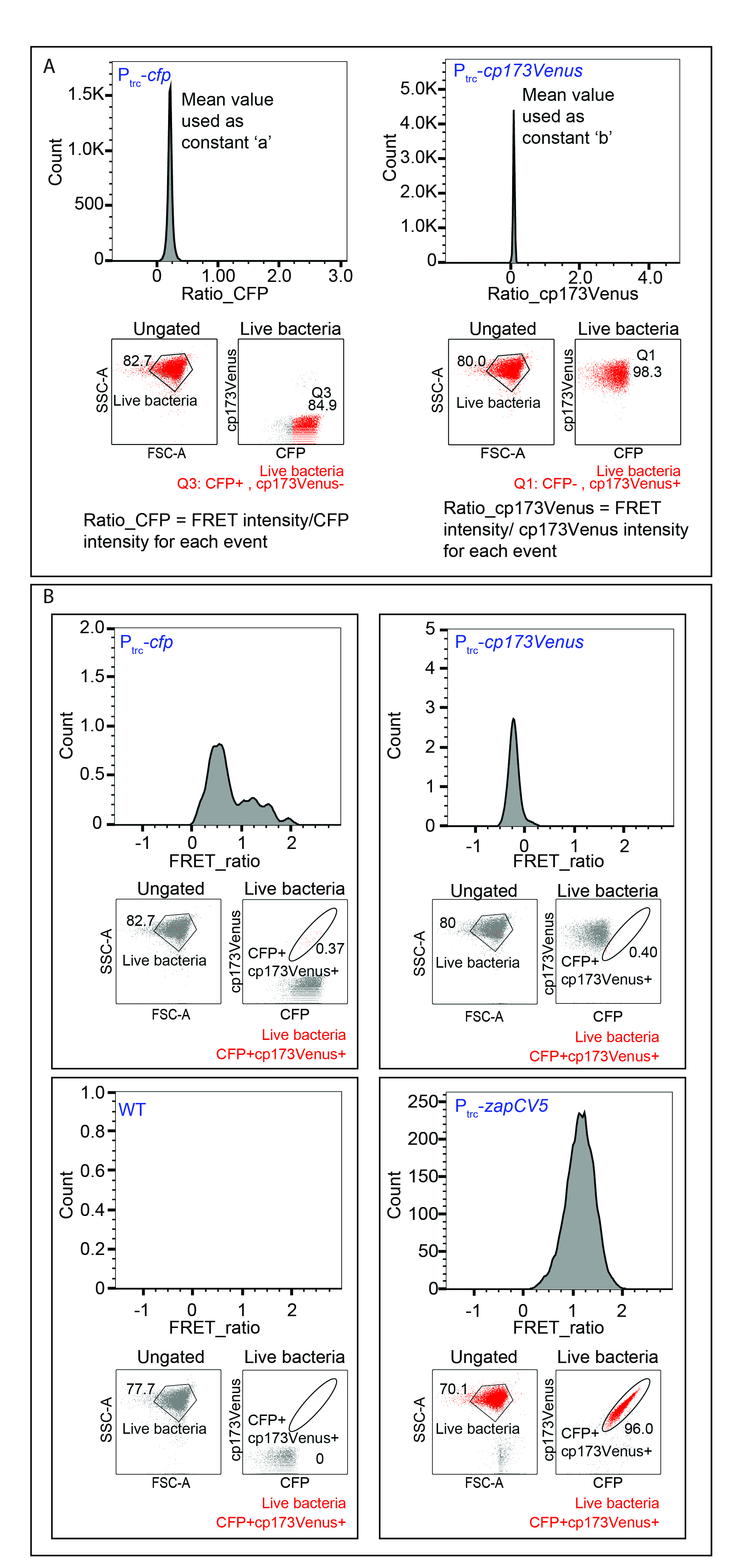

Supplement: FIG S7 [file mbo004184024sf7.tif]
