# Supplementary material for: Nitric Oxide Disrupts Zinc Homeostasis in Salmonella enterica Serovar Typhimurium
Source: mBio. 2018 Aug 14;9(4):e01040-18. doi: 10.1128/mBio.01040-18 (PMC6094482; doi:10.1128/mBio.01040-18)
Supplement: TABLE S2 [file mbo004184024st2.docx]

**Supplementary Table 2. Strains and plasmids**

**Plasmid or Strain Genotype Source**

| pKD46 | *bla araC-*P_araB_-γβ *exo* oriR101 repA101ts | Datsenko and Wanner 2000 |
| --- | --- | --- |
| pKD3 | *bla FRTcatFRT* PS1 PS2 oriRγ | Datsenko and Wanner 2000 |
| pKD4 | *bla FRTaphFRT* PS1 PS2 oriRγ | Datsenko and Wanner 2000 |
|  |  |  |
| pCP20 | *bla cat* cI857 IPr *flp* PSC101 oriTS | Datsenko and Wanner 2000 |
|  |  |  |
| pBAD18-Cm | *cat* P_araBAD_ oricolE1 | Guzman et al. 1995 |
|  |  |  |
| pRB3-273C | *bla par* RK2 oriV *trfA* | Berggren et al. 1995 |
|  |  |  |
| pHR103 | *bla* Δ*LacIQ* oricolE1 P_trc_ | Frawley et al. 2013 |
|  |  |  |
| pJBA111 | *bla pUC18Not-P_A1/04/03_-RBSII-gfp(LVA)-T_0_-T_1_* | Andersen et al. 1998 |
|  |  |  |
| pJK682 | *bla pBR322 RBSII gfp* | This study |
|  |  |  |
| pJK715 | *cat* P_araBAD_-*glyA* oricolE1 | This study |
|  |  |  |
| pJK719 (pZntA) | *bla par* RK2 oriV *trfA* P_zntA_-*zntA* | This study |
|  |  |  |
| pJK720 (pZntB) | *bla par* RK2 oriV *trfA* P_zntB_-*zntB* | This study |
|  |  |  |
| pJK721 (pZitB) | *bla par* RK2 oriV *trfA* P_zitB_-*zitB* | This study |
|  |  |  |
| pcDNA3.1-zapCV5 | *bla* *neo* oricolE1 orif1 P_CMV_-*zapCV5* | Fiedler et al. 2017 |
|  |  |  |
| pAS3 | *bla* P_araBAD_-*zapCV5* | This study |
|  |  |  |
| pAS4 | *bla* P_araBAD_-*cfp* | This study |
|  |  |  |
| pAS5 | *bla* P_araBAD_- *cpVenus* | This study |
|  |  |  |
| pAS15 | *bla* Δ*LacIQ* oricolE1 P_trc_-*zapCV5* | This study |
|  |  |  |
| pAS16 | *bla* Δ*LacIQ* oricolE1 P_trc_-*cfp* | This study |
|  |  |  |
| pAS17 | *bla* Δ*LacIQ* oricolE1 P_trc_-*cpVenus* | This study |
|  |  |  |
|  |  |  |
| pRU001 | *bla pBR322 RBSII gfp P_rpsm_-mCherry* | This study |
|  |  |  |
| pAS20 | *bla pBR322 RBSII P_hmp_-gfp P_rpsm_-mCherry* | This study |
|  |  |  |
| pAS22 | *bla pBR322 RBSII P_zntA_-gfp P_rpsm_-mCherry* | This study |
|  |  |  |
| BC1459 | Δ*fljBA::frt-kan-frt* | Cookson Lab, University of Washington |
|  |  |  |
| TH6726 | *fliC5569::tetRA* (+1 UTR) | Aldridge et al. 2006 |
|  |  |  |
| JK237 | 14028s | ATCC |
|  |  |  |
| JK377 | 14028s *fliC5569::tetRA*(+1 UTR) Δ*fljBA::frt-kan-frt* | This study |
|  |  |  |
| JK895 (FLS187) | 14028s/pRB3-273C | Richardson et al. 2011 |
|  |  |  |
| JK1284 | 14028s/pBAD18-Cm | This study |
|  |  |  |
| JK1285 | 14028s/pJK715 | This study |
|  |  |  |
| EF487 | Δ*zntA::frt-cm-frt* | This study |
|  |  |  |
| EF511 | Δ*zntB::tetRA* | This study |
|  |  |  |
| EF512 | Δ*zitB::frt-kan-frt* | This study |
|  |  |  |
| EF527 | Δ*zntA::frt-cm-frt* Δ*zntB::tetRA* | This study |
|  |  |  |
| EF528 | Δ*zntA::frt-cm-frt* Δ*zitB::frt-kan-frt* | This study |
|  |  |  |
| EF529 | Δ*zntB::tetRA* Δ*zitB::frt-kan-frt* | This study |
|  |  |  |
| EF530 | Δ*zntA::frt-cm-frt* Δ*zntB::tetRA* Δ*zitB::frt-kan-frt* | This study |
|  |  |  |
| EF531 | Δ*zntA::FRT* Δ*zntB::tetRA* | This study |
|  |  |  |
| EF532 | Δ*zntA::FRT* Δ*zitB::FRT* | This study |
|  |  |  |
| EF533 | Δ*zntB::tetRA* Δ*zitB::FRT* | This study |
|  |  |  |
| EF534 | Δ*zntA::FRT* Δ*zntB::tetRA* Δ*zitB::FRT* | This study |
|  |  |  |
| EF535 | Δ*zntA::frt-cm-frt*/pRB3-273C | This study |
|  |  |  |
| EF536 | Δ*zntA::frt-cm-frt*/pJK719 | This study |
|  |  |  |
| EF539 | Δ*zntA::FRT* Δ*zntB::tetRA*/pRB3-273C | This study |
|  |  |  |
| EF540 | Δ*zntA::FRT* Δ*zntB::tetRA*/pJK719 | This study |
|  |  |  |
| EF543 | Δ*zntA::FRT* Δ*zitB::FRT*/pRB3-273C | This study |
|  |  |  |
| EF544 | Δ*zntA::FRT* Δ*zitB::FRT*/pJK719 | This study |
|  |  |  |
| EF545 | Δ*zntA::FRT* Δ*zitB::FRT*/pJK721 | This study |
|  |  |  |
| EF548 | Δ*zntA::FRT* Δ*zntB::tetRA* Δ*zitB::FRT*/pRB3-273C | This study |
|  |  |  |
| EF549 | Δ*zntA::FRT* Δ*zntB::tetRA* Δ*zitB::FRT*/pJK719 | This study |
|  |  |  |
| EF550 | Δ*zntA::FRT* Δ*zntB::tetRA* Δ*zitB::FRT*/pJK721 | This study |
|  |  |  |
| EF553 | Δ*zntA::FRT* Δ*zntB::tetRA*/pJK720 | This study |
|  |  |  |
| EF561 | Δ*yiiP::frt-cm-frt* | This study |
|  |  |  |
| EF562 | Δ*yiiP::frt-cm-frt* Δ*zntA::FRT* Δ*zitB::FRT* | This study |
|  |  |  |
| EF563 | Δ*yiiP::frt-cm-frt* Δ*zntA::FRT* Δ*zntB::tetRA* Δ*zitB::FRT* | This study |
|  |  |  |
| AS168 | 14028s/pAS15 | This study |
|  |  |  |
| AS169 | 14028s/pAS16 | This study |
|  |  |  |
| AS170 | 14028s/pAS17 | This study |
|  |  |  |
| AS172 | Δ*zntA::FRT* Δ*zitB::FRT*/pAS15 | This study |
|  |  |  |
| AS212 | 14028s/pAS20 | This study |
| AS214 | 14028s/pAS22 | This study |
|  |  |  |
|  |  |  |
|  |  |  |
|  |  |  |
|  |  |  |
|  |  |  |
|  |  |  |
|  |  |  |
|  |  |  |
|  |  |  |
|  |  |  |
