# Supplementary material for: Nitric Oxide Disrupts Zinc Homeostasis in Salmonella enterica Serovar Typhimurium
Source: mBio. 2018 Aug 14;9(4):e01040-18. doi: 10.1128/mBio.01040-18 (PMC6094482; doi:10.1128/mBio.01040-18)
Supplement: TABLE S3 [file mbo004184024st3.docx]

**Supplementary Table 3. Primer sequences**

**Primer Sequence 5’-3’ Purpose**

| EFP227 | GGAGGACGTTATGTCGACCCCTGATACCGATGGCAAAAATGTGTAGGCTGGAGCTGCTTC | Deletion of *zntA* |
| --- | --- | --- |
|  |  |  |
| EFP228 | GAGAGGATTAGTTTTTACGCAAACGTAGCGCGTTGCACATATGAATATCTCCTTAG | Deletion of *zntA* |
|  |  |  |
| EFP235 | TGGAAGCCATTAAGGGATCGGATGTGAACGTGCCGGATGCTTAAGACCCACTTTCACATT | Deletion of *zntB* |
|  |  |  |
| EFP236 | CCATTTACTGCGATGCAACCATAAAGTAACACCACCGATCCTAAGCACTTGTCTCCTG | Deletion of *zntB* |
|  |  |  |
| EFP237 | ATGGCGCACTCACACTCTCATGCCGATTCACACCTGCGGAGTGTAGGCTGGAGCTGCTTC | Deletion of *zitB* |
|  |  |  |
| EFP238 | TTAATGGTGATGAACATGCCCGGACGATGTCTGATTCAGACATATGAATATCCTCCTTAG | Deletion of *zitB* |
|  |  |  |
| fljB-frtstart | ATAACATTGGTTATCAAAAACCTTCCAAAAGGAAAATTTTTGTGTAGGCTGGAGCTGCTTC | BC1459 construction |
|  |  |  |
| 3’fljA-FRT | CTTTTCTCACGGAATTTTTTATTACCGTAGGCGCATATGAATATCCTCCTTAG | BC1459 construction |
|  |  |  |
| JKP346 | AACCGCTAGCGAATTCAAATTTGGTACCAGAATTAAAGAGGAGAAATTAACGATGCGTAAAGGAGAAGAACT | pJK682 construction |
|  |  |  |
| JKP347 | AACCGACGTCTTATTTGTATAGTTCATCCATGC | pJK682 construction |
|  |  |  |
| JKP351 | GGCGGCCAAAGCGGTCGGA | pAS20, pAS22 confirmation |
|  |  |  |
| JKP356 | TGCCCATTAACATCACCATC | pAS20, pAS22 confirmation |
|  |  |  |
| JKP601 | AACCGCTAGCAGGAGGATTCACCATGTTAAAGCGTGAAATGACC | pJK715 construction |
|  |  |  |
| JKP602 | AACCAAGCTTATGCGTAAACCGGGAAGC | pJK715 construction |
|  |  |  |
| JKP717 | AACCGGATCCATAATAGCGCTTGACTCTGG | pJK719 construction |
|  |  |  |
| JKP718 | AACCAAGCTTGCCGGGCGGGAGAAGATTAG | pJK719 construction |
|  |  |  |
| JKP719 | AACCGGATCCATACATGTTTGGTAAGCGCC | pJK720 construction |
|  |  |  |
| JKP720 | AACCAAGCTTTGTTACAACCATTTACTGCG | pJK720 construction |
|  |  |  |
| JKP721 | AACCGGATCCCGGTAATGGCGTCAATAATG | pJK721 construction |
|  |  |  |
| JKP722 | AACCAAGCTTTCTGATCCTGACATTTATCG | pJK721 construction |
|  |  |  |
| JKP744 | CCTATGGACGGCTGGTTAGCCGGGCGGCTATCGCGGCAACGTGTAGGCTGGAGCTGCTTC | Deletion of *yiiP* |
|  |  |  |
| JKP745 | AATGACATCTGAACCCGGAAAACGCTGTAAAATCGCCTGCCATATGAATATCTCCTTAG | Deletion of *yiiP* |
|  |  |  |
| ASP21 | CTTCCAGGGGGATCCCATGGTGAGC | pAS4 construction |
|  |  |  |
| ASP22 | GTCATTGTTTGAATTCTAGCGGGCGGCGG | pAS4 construction |
|  |  |  |
| ASP23 | CATGGTATGGATCCCATGGACGGCGGCG | pAS5 construction |
|  |  |  |
| ASP24 | CCAAGCTTCGAATTCTTACTCGATG | pAS5 construction |
|  |  |  |
| ASP29 | TTTTGTCTAGATTTAAGAAGGAGATATACATATG | pAS15,16,17 construction |
|  |  |  |
| ASP30 | CCAAAACAGCCAAGCTTCGAATTC | pAS15,16,17 construction |
|  |  |  |
| ASP41 | GACGAATTCCAACGGATTTCTTTTC | pAS20 construction |
|  |  |  |
| ASP42 | GGGGAATTCTGGCCTTTACTGTAGCG | pAS20 construction |
|  |  |  |
| ASP45 | CCGCGAATTCCGGGCGGATAACGCG | pAS22 construction |
|  |  |  |
| ASP46 | CGGAATTCTGTATTGCCGGGGCTAATC | pAS22 construction |
